# Dose-Response Relationship of a Web-Based Tailored Intervention Promoting Human Papillomavirus Vaccination: Process Evaluation of a Randomized Controlled Trial

**DOI:** 10.2196/14822

**Published:** 2020-07-17

**Authors:** Mirjam Pot, Theo GWM Paulussen, Robert AC Ruiter, Liesbeth Mollema, Miranda Hofstra, Hilde M Van Keulen

**Affiliations:** 1 Child Health Netherlands Organization for Applied Scientific Research (TNO) Leiden Netherlands; 2 Department of Work and Social Psychology Maastricht University Maastricht Netherlands; 3 National Institute for Public Health and the Environment (RIVM) Centre for Infectious Disease Control Bilthoven Netherlands

**Keywords:** HPV vaccination, web-based tailored intervention, process evaluation, intervention use, dose-response relationship, randomized controlled trial, informed decision making

## Abstract

**Background:**

In the Netherlands, human papillomavirus (HPV) vaccination uptake remains low. To improve informed decision making (IDM) and HPV vaccination acceptability, we systematically developed an interactive, web-based tailored intervention to which mothers of Dutch girls were invited to participate.

**Objective:**

The aim of this study was to provide insight into the intervention’s working mechanisms by evaluating (1) program use, (2) program acceptability, and (3) the relationship of program use with program acceptability and intervention effects (ie, dose-response relationship).

**Methods:**

Only mothers from the intervention arm of a randomized controlled trial that assessed the effectiveness of the web-based, tailored intervention were included in this study. They were invited to visit the website of the web-based intervention between baseline (January 2015, just before access to the intervention) and follow up (March 2015, prior to the first HPV vaccination). Indicators for program use were time of website use (ie, duration of intervention exposure) and completeness (ie, proportion of all available web pages visited). HPV vaccination uptake registered by Praeventis was used as the primary outcome. Secondary outcomes were IDM, decisional conflict, and social-psychological determinants of HPV vaccination uptake.

**Results:**

Among the 3995 invited mothers, 2509 (62.80%) logged on to the website, 2239 of whom (89.24%) visited at least one page of the intervention components. On average, mothers spent 21.39 minutes (SD 12.41) on the website and completed 50.04% (SD 26.18%) of the website components. Participants rated the website 7.64 (SD 1.39) on a 10-point scale. Program acceptability was significantly associated with completeness (β=4.36, *P*<.001), but not with time of website use (β=–.07, *P*=.77). Intention-to-treat analysis (N=3995) showed a significant positive effect of completeness on all outcome measures (all *P*<.003; Bonferroni-corrected α=.05/15 factors), including on HPV vaccination uptake. Time of website use had a significant positive effect on all outcomes (all *P*<.003), except for uptake (*P*=.20), risk perception when not vaccinated (*P*=.14), subjective norms (*P*=.03), and habit (*P*=.01).

**Conclusions:**

Program use and acceptability of the intervention were adequate. Completeness was positively associated with acceptability. Furthermore, positive effects (ie, dose-response effects) were found for completeness and time of website use on the mothers’ IDM, decisional conflict, and almost all of the social-psychological determinants of HPV vaccination acceptability. In addition, the extent to which mothers completed the intervention had a positive impact on their daughters’ vaccination uptake. This indicates that the web-based, tailored intervention fits well with the mothers’ needs, and that completeness of use is essential for improving HPV vaccination uptake, acceptability, and IDM. Program use should therefore be promoted.

**Trial Registration:**

Netherlands Trial Register NTR4795; https://www.trialregister.nl/trial/4795

## Introduction

Cervical cancer is the fourth most common cancer among women worldwide. Globally, in 2018, there were 569,847 new cases and 311,365 deaths caused by cervical cancer [1]. Persistent infection by human papillomavirus (HPV) appears to be the major cause of cervical cancer; nearly all cases (over 99%) are attributable to an HPV infection [2,3]. There is also an increasing body of evidence that strongly links HPV infection with cancers of the anus, vulva, vagina, penis, and head and neck (eg, [4,5]). In the Netherlands, on an annual basis since 2010, girls are invited by the Dutch National Immunization Program to receive the vaccine against HPV upon reaching 13 years of age. However, HPV vaccination uptake remains low (45.5% in 2017) [6]. The uptake needs to be higher to reduce the incidence of cervical cancer in women. Therefore, we developed an interactive, web-based, tailored intervention promoting HPV vaccination acceptability among Dutch mothers of invited girls for HPV vaccination [7]. Further, to improve vaccine acceptability, important intervention objectives were to improve informed decision making (IDM) and to decrease decisional conflict [7].

To date, only four studies have examined the effectiveness of tailored interventions in promoting HPV vaccination acceptance [8-11]; three of these studies showed positive effects [9-11]. To our knowledge, the only tailored intervention that was web-based turned out to be ineffective [8]. The advantage of web-based interventions is that they have the potential to enable large-scale application at relatively low cost [12] and therefore can have a substantial impact at the population level [13].

Furthermore, only two of the existing studies using tailored interventions to promote HPV vaccination acceptance incorporated a process evaluation [9,11]. Process evaluation provides insight into the extent to which an intervention was used, which is relevant for determining factors that influence the effectiveness of an intervention [14-16]. Specifically, this analysis can provide insight into whether an intervention is ineffective due to implementation failure or to the inferior quality of the intervention method(s), thereby avoiding the so-called type III error [17]. It also provides recommendations for intervention improvement.

For the use of online interventions, three aspects are relevant: a first visit, staying, and revisiting [18,19]. In the context of decision making about HPV vaccination, we consider a first visit and staying as the most relevant aspects. Revisiting seems less relevant, since getting the HPV vaccine is hardly a repetitive behavior (ie, it consists of two injections). Within the electronic health (eHealth) arena, limited intervention use in terms of a first visit and staying is a broadly recognized problem [20]. The extent to which one uses an intervention may positively impact the intervention effects (ie, the so-called “dose-response relation”). Positive dose-response relationships have been found for eHealth interventions (eg, [21-23]). In the context of promoting HPV vaccination acceptability, none of the studies that incorporated a process evaluation addressed the dose-response relationship (for a review, see [24]). Moreover, data on the use of web-based interventions have been poorly reported [20,25] or not reported at all [26].

Furthermore, assessing program acceptability may shed light on the causes of nonuse or incomplete use [27]. An intervention needs to be considered appropriate by the target group and to suit their needs in order to reach the intended outcomes [28]. Acceptability will improve interaction and the users’ feelings of engagement, and, as a consequence, the likelihood of health-related behavior changes [29].

Results from the effect evaluation showed positive intervention effects on IDM, decisional conflict, and nearly all determinants of HPV vaccination uptake (for more details on the results of the effect evaluation, see [30]). This paper describes the process evaluation of the web-based tailored intervention promoting HPV vaccination acceptance [7]. The aim of this process evaluation was to provide insights into the intervention’s working mechanisms by evaluating (1) program use, (2) program acceptability, and (3) the relationship of program use with program acceptability and intervention effects (ie, dose-response relationships).

## Methods

### Participants and Study Design

The study was approved by the Ethical Committee of the VU Medical Center in Amsterdam (Netherlands Trial Register: NTR4795). This process evaluation was part of a randomized controlled trial on the effectiveness of the intervention [30]. For the purpose of this study, only participants from the experimental condition (ie, those invited to use the web-based intervention; N=3995) were included in the analyses. Details of the trial design, such as power calculations, participant eligibility, and recruitment procedures, have been published elsewhere [30]. This study was conducted between January 2015 (baseline, just before access to the intervention) and March 2015 (follow up, prior to the first HPV vaccination), in line with the national HPV vaccination round of 2015. Mothers were invited to use the web-based intervention between baseline and follow up (mid-January 2015).

### Intervention Components

We developed the web-based tailored intervention with virtual assistants using the 6-step Intervention Mapping protocol for developing theory- and evidence-based health promotion interventions [31]. In short, the web-based intervention provided mothers with tailored feedback about topics on HPV vaccination, delivered by two virtual assistants. Multimedia Appendix 1 presents a selection of screenshots of the website. The website consisted of 4 menu options: (1) 2-sided, tailored information about HPV vaccination, (2) a decisional balance in which mothers could weigh their perceived pros and cons, (3) practical information, and (4) frequently asked questions. Within menus 1-3, mothers could visit several components. In addition, mothers were given the opportunity to visit an “in-depth” information page within some of the components (eg, educational movies). Table 1 provides a brief overview of the intervention components. More detailed descriptions of the intervention, including its theoretical basis, are published elsewhere [7].

**Table 1:** Brief description of intervention components.

Menu and component		Description
**Information about HPV^a^ vaccination**		
	General information	Mothers are provided with general information about HPV, cervical cancer, and HPV vaccination.
	Importance of vaccinating at a young age	Mothers are challenged to consider whether the age of their daughter is appropriate to get vaccinated against HPV. The relationship between sexual activity in relation to the HPV vaccine is also discussed.
	HPV-related risks	Mothers are asked to estimate both the risk of their daughter getting infected with HPV and the risk of their daughter developing cervical cancer later in life, and are then provided with tailored feedback accordingly.
	Methods to protect against cervical cancer	Mothers are asked to rate the effectiveness of alternative methods for protecting against cervical cancer and are then provided with tailored feedback according to their answers.
	From HPV to cervical cancer	Mothers are provided with an explanation of how infection with HPV can lead to cervical cancer (eg, by viewing an educational video).
	Facts and stories	Mothers are provided with several statements regarding HPV, cervical cancer, and HPV vaccination, and are asked to indicate whether these are true (a fact) or false (a story). They then receive tailored feedback accordingly.
	Side effects	Mothers are presented with a variety of potential side effects of the HPV vaccine and are asked to indicate whether or not they are scientifically proven. They are then provided with tailored feedback, stating the correct responses.
	Effectiveness	Mothers are asked about the effectiveness of the HPV vaccine in protecting both against their daughter getting infected with HPV and developing cervical cancer. They are then provided with tailored feedback about the effectiveness of HPV vaccination.
	Other mothers	Mothers are asked to indicate what they think most mothers in their direct environment will decide regarding their daughters’ HPV vaccination. They are then provided with tailored feedback and are shown the actual HPV vaccination uptake in different regions of the Netherlands in 2014.
	Vaccine working mechanisms	Mothers are explained in a generic way how the HPV vaccine works in protecting against HPV and cervical cancer with an educational video.
**Weighing the pros and cons**		
	Decisional balance	Mothers are presented with a list of pros and cons of HPV vaccination. Based on pros and cons mothers marked as most salient, a decisional balance reveals their current position on a scale ranging between wanting and not wanting to get their daughter vaccinated.
	Value clarification	Mothers are invited to list their central values for life and link these to HPV vaccination.
Practical information		Practical information provides mothers with information such as how and where to receive the HPV vaccine and provides them with advice on how they discuss HPV vaccination with personal important others (eg, their daughter and partner).
**Frequently asked questions**		
	About HPV vaccination	Frequently asked questions provides answers to known questions about the HPV vaccine (eg, “does my daughter know if she’s infected with HPV?”) or getting the HPV vaccine (eg, “where do I get the HPV vaccine?”).
	Problems with the website	Mothers are provided with possible solutions to problems with the website such as not being able to hear or see the virtual assistants.

^a^HPV: human papillomavirus.

### Measurements

All data were derived from web-based self-report questionnaires and computer logs.

### Program Use

Program use was measured according to time of website use and completeness. Both factors were constructed based on computer logs that registered the pages and components the mothers had visited and the amount of time they spent on the website during their visit(s).

Time of website use represents the total amount of time spent on the website. Each time mothers logged on to the website, a record was created, in which both the date and time of the first and last page the mothers entered were registered. Time per session was calculated by subtracting the time of the last entered page from the time of the earliest entered page. The total time of website use was then calculated by adding up the amount of time spent in each session.

Completeness represents the proportion of all available web pages visited by the mother while she was logged on to the website, ranging from 0% (visited no pages) to 100% (visited all pages).

### Program Acceptability

Program acceptability was assessed at the 6-week follow up by asking mothers to rate the intervention and the virtual assistants on a 10-point scale, ranging from 0 (very bad) to 10 (excellent). The grade for the website was used as an indicator for overall program acceptability to examine the association with program use. In addition, mothers evaluated the information provided by the website (eg, credibility, relevance), perceived user control (eg, perceived degree of autonomy), and how they perceived the virtual assistants (eg, reliability) (See Table 2 and Table 3 for an overview). These acceptability measures were assessed on a 7-point scale, and some were averaged into one scale. All composite scores showed sufficient internal consistency (Cronbach α ≥.71 for scales with >2 items; Pearson *r*≥0.84 for scales with 2 items).

**Table 2:** Overview of the program acceptability measures for the website.

Measures	Items	Score and scale	Cronbach α or Pearson *r*	Reference
Rate	On average, how would you rate the website on a scale from 0 to 10?	0=very bad to 10=excellent	N/A^a^	[32]
Interest	In general, what did you think of the website?	1=very uninteresting to 7=very interesting; 1=boring to 7=engaging	0.84 (*r*)	[33]
Informative	In general, what did you think of the website?	1=very uninformative to 7=very informative; 1=very noneducational to 7 =very educational	0.86 (*r*)	[34]
Perceived user control	I felt that I had a lot of control over my visiting experiences at this website. While I was on the website, I could choose freely what I wanted to see. While surfing the website, I felt in control. While surfing the website, my actions decided the kind of experiences I got.	1=strongly disagree to 7=strongly agree	.71 (*α*)	[35]
Elaboration	How well did you read the information?	1=not carefully at all to 7=very carefully	N/A	[36]
Support	The website has helped me decide about my daughter’s HPV^b^ vaccination.	1=strongly disagree to 7=strongly agree	N/A	[37]
Recall	I can recall the information from the website.	1=strongly disagree to 7=strongly agree	N/A	[32]
Personal relevance	I considered the website to be personally relevant.	1=strongly disagree to 7=strongly agree	N/A	[32,37]
Tailoring	I considered the information on the website to be…	1=not tailored to me at all to 7=very tailored to me	N/A	[37]
Comprehensibility	I considered the information on the website to be…	1=not at all understandable to 7=very understandable	N/A	[37]
Reliability	I considered the information on the website to be…	1=very unreliable to 7=very reliable	N/A	[37]
Credibility	I considered the information on the website to be…	1=very incredible to 7=very credible	N/A	[37]
Usefulness	I considered the information on the website to be…	1=very useless to 7=very useful	N/A	[38]
Readability	I considered the information on the website to be…	1=very unreadable to 7=very readable	N/A	[39]
Sidedness	I considered the information on the website to be…	1=focused on the cons to 7=focused on the pros	N/A	[40]
Enjoyment	I considered the information on the website to be…	1=very unenjoyable to 7=very enjoyable	N/A	[41]
Novelty	The website contained new information for me.	1=strongly disagree to 7=strongly agree	N/A	[39]
Attitude toward website	How good or bad did you find… - the possibility to first answer a question and then receive information? - the possibility to weigh the pros and cons? - the speed of the website? - the layout of the website?	1=very bad to 7=very good	.81 (*α*)	[42]

^a^N/A: not applicable.

^b^HPV: human papillomavirus.

**Table 3:** Overview of the program acceptability measures for the virtual assistants.

Measures	Items	Score and scale	Pearson *r*	Reference
Overall rating	On a scale from 0 to 10, how would you rate: - the mother-like assistant - the doctor-like assistant	0=very bad to 10=excellent	0.94	[43]
Enjoyment	I considered the virtual assistants to be…	1=very unenjoyable to 7=very enjoyable	N/A^a^	[41]
Reliability	I considered the virtual assistants to be…	1=very unreliable to 7=very reliable	N/A	[43]
Credibility	I considered the virtual assistants to be…	1=very incredible to 7=very credible	N/A	[37]
Usefulness	I considered the virtual assistants to be…	1=very useless to 7=very useful	N/A	[37]

^a^N/A: not applicable.

### Outcomes

#### Primary Outcome: HPV Vaccination Uptake

An objective measure for HPV vaccination uptake was derived from Praeventis, which is the national electronic vaccination register that monitors the vaccination status for all children up to 18 years of age living in the Netherlands [44]. Uptake was dichotomized into having received no HPV injection (0=not vaccinated) and having received 1 or 2 HPV injections (1=vaccinated), as data analyses showed that determinants of HPV vaccination uptake contrasted the most between these groups.

#### Secondary Outcomes: IDM, Decisional Conflict, and Social-Psychological Determinants of HPV Vaccination Uptake

Multimedia Appendix 2 provides the measurement details of the constructs IDM, decisional conflict, and the social-psychological determinants of HPV vaccination uptake [30,45-54]. Determinants accounted for were intention, attitude, risk perception of having received the HPV vaccine, risk perception without having received the HPV vaccine, anticipated regret about rejecting the HPV vaccine, beliefs, subjective norms, habit strength toward HPV vaccination, self-efficacy, knowledge, and perceived relative effectiveness of HPV vaccination compared to alternative methods.

### Sociodemographics

Sociodemographics were included as background variables (ie, age, educational level, country of birth, and religion).

Level of education referred to the mothers’ highest completed level of education. It was classified as low (less than secondary or vocational education), intermediate (secondary through preuniversity education), or high (professional or university education) [30,49,50].

Country of birth was dichotomized into “Netherlands” vs “other,” because the percentage of mothers born in the latter category (276/3995, 6.91%) was too small for further subdivision in our sample.

Religion was measured by asking mothers about their religious convictions (Protestant, Roman Catholic, Muslim, Jewish, Buddhist, Hindu, other, or no religion). This was dichotomized into “Protestant” vs “not Protestant” as earlier research and data analyses showed that Protestants more frequently refrain from vaccination compared to other religious groups [30,49,50].

### Statistical Analyses

The sample and intervention use data are summarized using descriptive statistics. For determining the dose-response relation, intention-to-treat (ITT) analyses were used, which increases power, while decreasing the risk of possible bias caused by selective dropout [55]. Missing data were imputed for uptake, determinants, and sociodemographics (not for use): we applied multiple imputation by chained equations [55,56]. In total, 15 imputed datasets were generated using the predictive mean matching algorithm in Statistical Package for the Social Sciences (SPSS, IBM Corp). Results from these datasets were pooled together using Rubin’s rules [57]. Iteration plots were inspected to check convergence of the imputations.

The relationship between acceptability and use was examined using univariate regression analyses. The dose-response relation between program use and intervention effects was examined by logistic and linear regression analyses (for dichotomous and continuous variables, respectively) using the outcome score at follow up as the dependent variable and the outcome scores at baseline and program use (both completeness and time of website use) as the independent variables [58]. Effects were considered significant when the *P* value was lower than .003 (Bonferroni-corrected α=.05/15 factors). For the logistic regressions, the odds ratio (OR) was used as an index of effect sizes. These were interpreted as small (OR=1.5), medium (OR=3.5), or large (OR=9.0) [59]. For the linear regressions, effect sizes were calculated in R software (R Development Core Team, Vienna, Austria) using Cohen ƒ^2^ statistic, (R^2^*AB* – R^2^*A*)/(1 – R^2^*AB*), in which *B* is the variable of interest (ie, use), *A* is the set of all other variables (ie, the outcome at baseline), R^2^*AB* is the proportion of variance accounting for *A* and *B* together, and R^2^*A* is the proportion of variance accounted for by *A* [60]. These were classified as small (ƒ^2^=0.02), medium (ƒ^2^=0.15), and large (ƒ^2^=0.35) [59]. We performed complete case analyses as a sensitivity check for substantial differences with the results from ITT.

## Results

### Response Rates and Attrition

**Figure 1:**
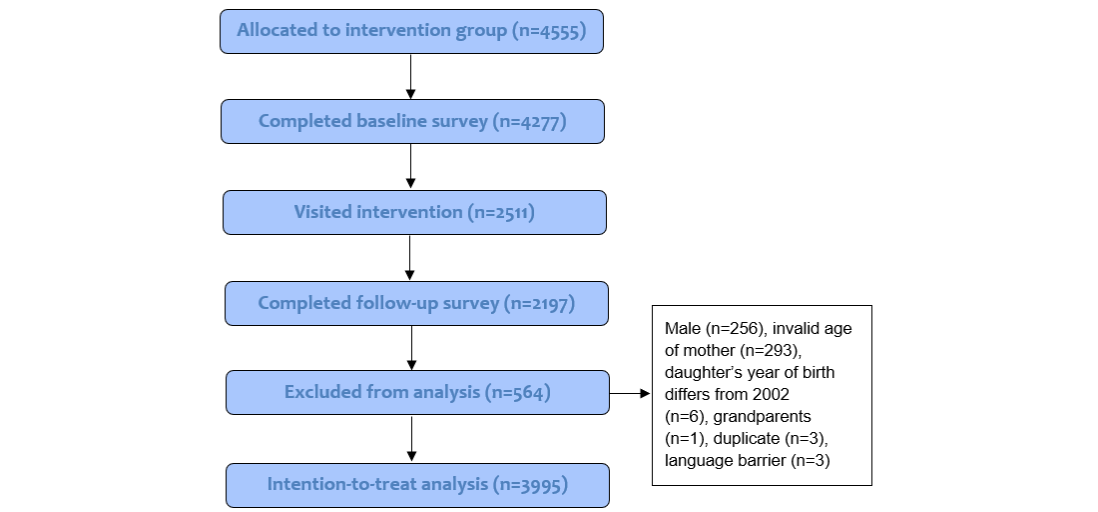
Flow diagram of the recruitment and response of study participants from the experimental condition. Participants could be excluded based on multiple criteria (eg, male with an invalid age). Therefore, the total number of participants excluded differed from the sum of separate criteria for exclusion.

A flow diagram of the recruitment and response of participants in the experimental condition, as part of the process evaluation, is shown in Figure 1. From the 4555 participants who were randomized into the experimental condition at baseline (T0), 4277 (93.90%) completed the baseline questionnaire, 2511 (55.13%) visited the intervention, and 2197 (48.23%) completed the follow-up questionnaire 8 weeks later (T1). Dropout analysis showed that there was significantly more dropout with regard to participants not born in the Netherlands, those with a lower educational level, those having a daughter not being vaccinated, and those with low levels of risk perception. In total, 564 participants were excluded as they (a) did not meet the inclusion criteria (ie, being a mother of a daughter born in 2002 and aged 24-62 years), (b) were found to be duplicates, or (c) encountered a language barrier. The final sample for ITT analysis consisted of 3995 mothers.

### Sample Description

Table 4 shows the sample description of participants in the experimental condition. No data were available on sociodemographics of the population from which the sample was derived (ie, Dutch mothers of 12-year-old girls); hence, we were unable to assess whether the study sample was representative. The mothers’ mean age was 43.70 years (range 27.00-62.00 years). On average, mothers had a positive intention toward their daughters’ HPV vaccination at baseline (mean score 5.35, SD 1.69). Compared with the national HPV vaccination uptake at the time (ie, 2015), uptake was higher in the study sample (59,866/93,173; 60.98% vs 2923/3995; 73.17%) [61].

**Table 4:** Sample description of mothers in the intervention group (N=3995).

Variables		N (% missing)	Value^a^
Age in years, mean (SD)		3995 (0)	43.70 (4.27)
**Country of birth, n (%)**		3991 (0.10)	
	The Netherlands		3715 (92.99)
	Other		276 (6.91)
**Religion, n (%)**		3998 (0.18)	
	Protestant		753 (18.85)
	Not Protestant		3235 (80.98)
**Educational level, n (%)**		3991 (0.10)	
	Low		588 (14.72)
	Middle		1736 (43.45)
	High		1660 (41.55)
**HPV^b^ vaccination uptake, n (%)**		3986 (0.23)	
	Yes		2923 (73.17)
	No		1063 (26.60)

^a^By reporting 2 decimal points for the percentages, summing the percentages for each category may differ from 100%.

^b^HPV: human papillomavirus.

### Program Use

From the 3995 mothers in the intervention group, a total of 2509 mothers (62.80%) logged in (ie, time of website use >0). Of these, 73.06% (1833/2509) logged in once, 19.89% (499/2509) logged in twice, 5.00% (125/2509) logged in three times, and 2.07% (52/2509) logged in four times or more. On average, mothers spent 21.39 minutes (SD 12.41) on the website.

Of the 2509 mothers that had initially logged in, 2239 (89.24%) visited at least one page of a component of the intervention (ie, completeness >0). Only these 2239 mothers were included for describing use of specific intervention components and program acceptability (see below). On average, the mothers completed 50.04% (SD 26.18%) of the website components (ie, 51/101 pages).

### Use of Intervention Components

Table 5 shows the breakdown for the use of intervention components (N=2239). When mothers clicked on one or more links to receive in-depth information, it was marked as “visited.” Mothers visited the page “Ways to Protect Against Cervical Cancer” the most frequently (1971/2239, 88.03%), followed by “Chance” (1945/2239, 86.87%), whereas they visited “Value Clarification” the least (293/2239, 12.53%). “General Information” had the highest completion rate (1622/2239, 72.44%), whereas “Side Effects” and “Effectivity” were completed the least (both 19/2239, 0.85%). Only a small percentage of the mothers (4.20%-13.22%; n=94-298) visited in-depth information (comprising educational movies or extra information).

**Table 5:** Program use of the various intervention components among those who visited at least one page of the intervention (N=2239).

Intervention component	Completed^a^, n (%)^b^	Partly completed^c^, n (%)	Not visited^d^, n (%)	Visited in-depth information^e^, n (%)
General Information	1622 (72.44)	71 (3.17)	546 (24.39)	N/A^f^
Ways to Protect against Cervical Cancer	130 (5.81)	1841 (82.22)	268 (11.97)	N/A
Chance	135 (6.03)	1810 (80.84)	294 (13.13)	142 (6.34)
From HPV^g^ to Cervical Cancer	96 (4.29)	1110 (49.58)	1033 (46.14)	298 (13.22)
Age	154 (6.88)	1568 (70.03)	517 (23.09)	160 (7.15)
Side Effects	19 (0.85)	1686 (75.30)	534 (23.85)	299 (13.35)
Effectivity	19 (0.85)	1542 (68.87)	678 (30.28)	339 (15.14)
Other Mothers	1099 (49.08)	416 (18.58)	724 (32.34)	N/A
Working Mechanisms Vaccination	94 (4.20)	1015 (45.33)	1130 (50.47)	94 (4.20)
Facts and Stories	95 (4.24)	1220 (54.49)	924 (41.27)	99 (4.42)
Weighing Pros and Cons	615 (27.47)	911 (40.69)	713 (31.84)	N/A
Value Clarification	269 (12.01)	24 (1.07)	1946 (86.91)	N/A
Practical Information	556 (24.83)	1147 (51.23)	536 (23.94)	N/A

^a^A component was considered “completed” when mothers visited every page of the component.

^b^By reporting 2 decimal points for the percentages, summing the percentages for each category may differ from 100%.

^c^“Partly completed” indicates that the mothers had seen at least one, but not all pages.

^d^“Not visited” means that the mothers had not visited any of the component’s pages.

^e^“Visited in-depth information” was also part of the total completeness, but is depicted separately here to provide a better overall view of the mothers’ interest in this information. When one or more of the links to more in-depth information had been clicked on, the in-depth information was marked as “visited.”

^f^N/A: not applicable as the component did not contain in-depth information.

^g^HPV: human papillomavirus.

### Program Acceptability

The overall acceptability of the intervention was rated 7.64 (SD 1.39) on a 10-point scale (1513/2239, 67.57%). Acceptability was significantly and positively associated with completeness (β=4.36, *P*<.001); this did not account for time of website use (β=–.07, *P*=.77). The virtual assistants were rated 7.41 (SD 1.57) on a 10-point scale (1513/2239, 67.57%). The mean scores on all other program acceptability measures were moderate to high (range 4.34-5.71 on a 7-point Likert scale; see Table 6). As the data were not normally distributed but were positively skewed, the median scores are also included in Table 6.

**Table 6:** Mean (SD) and median scores on the program acceptability measures among those who visited at least one page of the intervention (N=2239)^a^.

Variable	Website (n_missing_=726-734)		Virtual assistants (n_missing_=960)	
	Mean (SD)	Median	Mean (SD)	Median
Rate (1-10)	7.64 (1.39)	8.00	7.41 (1.57)	8.00
Interest (1-7)	5.41 (1.05)	5.50	N/A^b^	N/A
Informative (1-7)	5.67 (1.04)	6.00	N/A	N/A
Attitude toward website (1-7)	5.53 (.89)	6.00	N/A	N/A
Perceived user control (1-7)	5.26 (.97)	5.50	N/A	N/A
Elaboration (1-7)	5.70 (1.14)	6.00	N/A	N/A
Tailoring (1-7)	5.22 (1.21)	5.22	N/A	N/A
Comprehensibility (1-7)	5.40 (1.70)	6.00	N/A	N/A
Reliability (1-7)	5.27 (1.23)	6.00	5.04 (1.09)	5.00
Credibility (1-7)	5.35 (1.21)	6.00	5.01 (1.22)	5.00
Usefulness (1-7)	5.52 (1.10)	6.00	4.83 (1.33)	5.00
Readability (1-7)	5.71 (1.04)	6.00	N/A	N/A
Sidedness (1-7)	4.74 (1.09)	4.00	N/A	N/A
Enjoyment (1-7)	4.70 (1.08)	5.00	4.72 (1.37)	5.00
Novelty (1-7)	5.11 (1.39)	5.00	N/A	N/A
Support (1-7)	4.34 (1.80)	5.00	N/A	N/A
Recall (1-7)	5.16 (1.22)	5.00	N/A	N/A
Personal relevance (1-7)	4.87 (1.41)	5.00	N/A	N/A

^a^A higher score represents a higher score on the program acceptability measure.

^b^N/A: not applicable.

### Dose-Response Relation Between Program Use and Intervention Outcomes

Table 7 provides an overview of the effects of program use (completeness and time of website use) on the primary and secondary outcomes according to the ITT analyses. Significant positive effects were found for completeness on all outcome measures (all *P*<.003). The more mothers had completed the intervention, the more likely they were to have their daughter vaccinated against HPV and make an informed decision, and were less likely to experience decisional conflict at follow up. In addition, positive effects of completeness were found with respect to all social-psychological determinants of HPV vaccination acceptability (eg, HPV vaccination intention, beliefs; see Table 7).

Time of website use had a positive effect on all outcomes (all *P*<.003), except for HPV vaccination uptake, risk perception when not vaccinated, subjective norms, and habit (Table 7). The more time mothers spent on the intervention, the more likely they were to make an informed decision, experience less decisional conflict, have a higher intention to vaccinate their daughter, have a more positive attitude, have more positive beliefs, have a lower risk perception of HPV vaccination, anticipate more feelings of regret about rejecting the HPV vaccine, report a higher relative effectiveness of the HPV vaccine, have higher self-efficacy expectations, and have more knowledge at follow up.

Effect sizes were small overall (see Table 7). Results from the complete case analyses were similar for both completeness and time of website use, except for a lack of effect of time of website use on intention.

**Table 7:** Effects of use on the outcome measures according to intention-to-treat analyses (N=3995).

Outcome^a^			Pretest, mean (SD)		Posttest, mean (SD)	Completeness			Time of website use		
						β (SEM)	Cohen ƒ^2^ or OR^b^		β (SEM)	Cohen ƒ^2^ or OR	
**Primary outcome: HPV^c^ vaccination uptake^d^**											
	Has received no HPV injection (reference)	N/A^e^		26.67% (1066)		N/A	N/A		N/A	N/A	
	Has received one or two HPV injections	N/A		73.32% (2929)		.004 (.001)^f^	1.004		.003 (.003)^g^	1.003	
**Secondary outcomes**											
	IDM^h^: Not informed (reference)	67.31% (2689)		42.53% (1699)		N/A	N/A		N/A	N/A	
	IDM: Informed	32.69% (1306)		57.47% (2296)		.014 (.001)^f^	1.014		.021 (.003)^f^	1.021	
	IDM: continuous (0-48)	18.69 (11.21)		25.85 (12.30)		.087 (.006)^f^	0.075		.122 (.013)^f^	.027	
	Decisional conflict (1-7)	4.33 (1.75)		5.38 (1.36)		.007 (.001)^f^	0.043		.011 (.002)^f^	.018	
	Intention (1-7)	5.35 (1.69)		5.59 (1.87)		.004 (.001)^f^	0.014		.005 (.002)^f^	.004	
	Attitude (1- 7)	5.18 (1.45)		5.37 (1.51)		.005 (.001)^f^	0.027		.007 (.001)^f^	.010	
	Beliefs (1-7)	4.19 (.73)		4.47 (.81)		.004 (.000)^f^	0.051		.005 (.001)^f^	.016	
	Risk perception; not vaccinated (1-7)	3.74 (0.98)		3.77 (1.08)		.002 (.001)^f^	0.003		.002 (.001)^i^	.001	
	Risk perception; vaccinated (1-7)	2.77 (1.07)		2.64 (1.10)		-.003 (.001)^f^	0.008		–.005 (.001)^f^	.005	
	Anticipated regret (1-5)	3.71 (1.25)		3.59 (1.31)		.002 (.001)^f^	0.007		.004 (.001)^f^	.004	
	Subjective Norm (–20-20)	5.88 (7.81)		7.25 (9.20)		.020 (.004)^f^	0.009		.018 (.008)^j^	.002	
	Habit (1-7)	4.28 (1.78)		4.51 (1.83)		.004 (.001)^f^	0.011		.004 (.002)^k^	.003	
	Relative effectiveness (1-10)	-1.97 (2.22)		-1.35 (2.27)		.013 (.001)^f^	0.051		.016 (.003)^f^	.015	
	Self-efficacy (1-7)	6.27 (.73)		6.29 (.75)		.003 (.000)^f^	0.022		.004 (.001)^f^	.007	
	Knowledge (–8-8)	4.40 (2.14)		5.75 (2.09)		.017 (.001)^f^	0.095		.024 (.002)^f^	.034	

^a^A higher score means a higher outcome (eg, more positive attitude) except for decisional conflict in which a higher score means less decisional conflict.

^b^OR: odds ratio; OR>1 means the higher the score on a factor, the higher the outcome of IDM and higher chance of the daughter being vaccinated; OR<1 means the higher the score on a factor, the lower outcome of IDM and lower chance of the daughter being vaccinated.

^c^HPV: human papillomavirus.

^d^HPV vaccination uptake was not assessed at baseline.

^e^N/A: not applicable.

^f^*P*<.003 (significant; Bonferroni: 0.05/15 factors).

^g^*P*=.20.

^h^IDM: informed decision making.

^i^*P*=.14.

^j^*P*=.03.

^k^*P=*.01.

## Discussion

### Principal Findings

The aim of this process evaluation was to examine (1) program use, (2) program acceptability, and (3) the relationship between program use and acceptability and intervention outcomes for the web-based tailored intervention about HPV vaccination.

### Program Use

Almost two thirds (62.80%) of the mothers who were invited to visit the intervention logged in to the intervention. This reach is adequate, and is comparable to that reported for other eHealth interventions (eg, [62]). However, it still leaves room for improvement to increase the reach by employing strategies such as arousing interest in this eHealth intervention. Support for this notion comes from Crutzen et al [63], who showed that arousing interest successfully increased the intention to visit a website about hepatitis A, B, and C virus and the likelihood of clicking on the link to visit the website. Interest was aroused in the invitation by challenging the potential visitor regarding their knowledge about hepatitis and it was emphasized that the website provided this information in a comprehensible manner. Not only could interest be aroused in the invitation for HPV vaccination for girls and their parents but also, for instance, via other channels by which the target group is reached (eg, the internet, social media). In addition, research has shown a positive recommendation by word of mouth to be an important trigger for visiting a web-based intervention for the first time [19]. Word-of-mouth recommendations could be encouraged by providing “tell-a-friend” services at the web-based intervention [19].

Of the mothers that logged in to the intervention, nearly all visited at least one component. However, a small portion of the mothers (10.76%) did not view any of the intervention’s content after having logged in. This is likely due to technical difficulties because at follow up, some mothers (320, 12.75%) indicated that they were not able to see or hear the virtual assistants.

On average, time of website use, indicating the time mothers spent with the intervention, was adequate (21.39 minutes). This is longer than that reported by Brouwer and colleagues [26] in their review of web-based, interactive healthy lifestyle interventions, in which intervention exposure time varied from less than 10 minutes to 10-20 minutes. However, the present exposure time was lower than found by Sanders and colleagues [64] for their interactive, web-based tailored intervention promoting colorectal cancer screening (33 minutes). This difference could be explained by the controlled setting of their study [64], in which participants arrived 60 minutes prior to a medical appointment and viewed the intervention in the waiting room in the presence of a research assistant.

In addition, the mean completeness of the intervention was 50.04%. This indicates that the intervention fits well with the mothers’ needs, which is also supported by the results regarding acceptability (see below). The mean completeness in this study is comparable to that found by Watts and colleagues [65], who reported a completeness of 49.09% for a web-based prostate cancer screening decision aid.

### Use of Intervention Components

When looking at exposure to intervention components, we found that mothers were most interested in the effectiveness of (alternative) methods to protect against cervical cancer (88.03% visited) and the risks of their daughter getting infected with HPV and developing cervical cancer (86.87% visited). These appear to be essential components of communication about HPV vaccination.

The “Value Clarification” component was visited the least overall (13.09%). This may be attributed to the fact that it was not easily found by participants: this page was only accessible once mothers completed the component “Weighing Pros and Cons.” Voncken-Brewster and colleagues [66] also found that participants overlooked a certain part of their intervention, and they successfully improved its visibility by repositioning it. In order to promote exposure to values clarification, we could enhance its visibility.

The “General Information” component was completed the most overall (72.24%). This is likely due to the fact that the mother-like virtual assistant recommended starting with this component when mothers first entered the main menu, and that it was brief (2 pages). “Side Effects” and “Effectivity” were completed the least (both 0.85%). This could be explained by the fact that these two components contained the most links to in-depth information. Only a small proportion of the mothers (≤13.22%) visited in-depth information.

### Program Acceptability

The mothers evaluated the website as positive, as shown by the overall acceptability (7.64/10) and the scores on all acceptability measures (mean scores ranging from 4.34 to 5.71 on a 7-point Likert scale). The virtual assistants were also well appreciated (7.41/10). This is likely because it matched with the mothers’ preferences for more interactive personalized feedback [40]. This adequate acceptability is similar to the findings of Paiva and colleagues [67] for a web-based tailored intervention to increase HPV vaccination among young adult women (3.27/4.0). We believe the high acceptability in the present study can be attributed to the systematic and user-centered development process of the intervention [7]. The target group was extensively involved throughout the development. Not only did we fine-tune the content of the intervention to the mothers’ preferences and requirements but this also was considered in the design of the website, which was chosen by the mothers.

Furthermore, we found a positive relationship between acceptability and completeness (*P*<.001). This association was also found by others in a web-based intervention for breast cancer [68-70]. This finding underlines the importance of an intervention to be considered appropriate by the target group.

### Effects of Program Use on Intervention Outcomes

In line with our expectations, completeness had a significant positive effect on all outcome measures (IDM, decisional conflict, social-psychological determinants of HPV vaccination uptake), including actual HPV vaccination uptake itself. Mothers who had completed more of the intervention were more likely to have their daughter vaccinated against HPV, and had higher levels of IDM and more positive scores on determinants of HPV vaccination acceptance. In particular, the effect on HPV vaccination uptake is an important finding, which was not found when simply contrasting the experimental to the control condition [30]. This stresses the importance of conducting a process analysis alongside such an effect evaluation. These results are very promising, considering the currently low HPV vaccination uptake rates in the Netherlands [6].

We believe that these positive effects can be attributed to the extensive tailoring throughout the intervention. Support for this notion comes from the study results indicating that mothers perceived the intervention to be well-tailored (5.22 on a 7-point scale). Not only did we tailor the content of the intervention to the mothers’ personal interest, it was also used to guide the mothers’ personal route through the intervention. The latter is likely to have improved the usability of the intervention. Moreover, the intervention accounted for tailoring on a variety of themes. For example, it considered perceived barriers similar to the approach taken by Gerend and colleagues [9] as well as other beliefs, attitudes, subjective norms, habits, relative effectiveness, anticipated regret, risk perception, self-efficacy, and knowledge.

In contrast to our expectations, time of website use had a positive effect on all outcomes (*P*<.003), except for risk perception when not vaccinated (*P*=.14), subjective norms (*P*=.03), habit (*P*=.01), and vaccination uptake (*P*=.20). The latter is also in contrast to findings of a previous study [64]. An explanation for this lack of effects may be the measurement we used for time of website use, which may be less reliable for measuring exposure. We measured the total time spent on the website, but this does not indicate the specific pages the mothers had visited and for how long. In addition, we were unable to determine if there were any timeouts during a session and what the duration of a timeout was, because the intervention was web-based. For instance, they could have been distracted in the home environment during website use, which may have influenced the measured time of website use and may also have caused the lack of effect of acceptability on time of website use. An alternative, more accurate, measure can be found within the domain of education, namely time on task [71]. Future studies are needed to examine whether time on task (instead of total time of website use) has a positive influence on uptake.

### Methodological Considerations

There are three methodological considerations. First, as mentioned above, the measurement of total time spent on the website seemed to be not entirely adequate to measure program use. In this study, completeness seemed to be a better indicator of use as it demonstrates the mother’s navigation through the website, whereas time of website use did not. In addition, within (almost) every component, mothers were first asked a question about an HPV-related topic. The page was marked as “completed” if they provided an answer, which was necessary to obtain tailored feedback. Within the decisional balance, completeness was calculated based on the mother’s answer to each statement. These are more complete indicators of actual use. Nevertheless, it is not an entirely complete indicator for use, since we could not measure whether they read generic information, listened to the tailored feedback from the virtual assistant, or saw a video. This may have influenced the dose-response effects found.

Second, considering the aim of initiating active processing of information, the positive effect found of completeness on habit may seem unwanted. However, at follow up, the mothers had already been exposed to the intervention, and were therefore likely to have actively processed the information. These results may indicate that the more mothers completed the intervention, the less they had to think about getting the HPV vaccine. The intervention may have helped them make a decision rather than inducing perceptions about HPV vaccination as something you take for granted, without thinking. In retrospect, the label “habit” appears to be misleading.

Third, the effect sizes found were small, which is in line with other web-based interventions targeting health behavior outcomes [72]. Despite this, we believe that even small effects are of relevance in public health as they become substantial at the population level. After all, in the Netherlands, approximately 100,000 girls are invited to receive the HPV vaccine on a yearly basis.

### Strengths and Limitations

The most important strength of this study is that we, to our knowledge, are the first within the field of HPV vaccination to conduct an extensive process evaluation. According to a review [24], the process evaluation of an intervention promoting HPV vaccination acceptance by tailoring information to participants’ perceived barriers, such as that performed by Gerend and colleagues [9], was limited to the evaluation of intervention information (eg, the extent to which the information was informative and convincing). Other notable strengths are the objective measurement of HPV vaccination uptake, the dose-response effect of completeness on actual HPV vaccination uptake, the broad focus on outcomes (ie, determinants of HPV vaccination acceptance, uptake, IDM, and decisional conflict), and the adequate level of both (objectively assessed) program use and acceptability.

However, some limitations need to be addressed. First, there appeared to be technical difficulties despite our best effort to minimize such issues (eg, by extensively pilot testing the website using various devices). For instance, it turned out that a portion of the mothers were unable to see or hear the virtual assistants and that certain components did not work adequately among some of the mothers. Such technical difficulties are likely to have influenced website usability and therefore may have had a negative impact on program use. Second, this eHealth intervention requires mothers to have eHealth literacy [73]. People with lower eHealth literacy have been shown to have a lower educational level and to spend less time online [74,75]. It could be interesting to examine the extent to which eHealth literacy might have influenced use, acceptability, and effects. Considering the latter, we do not expect substantial differences since we did not find any differences in intervention effects among mothers with a low vs high educational level [30]. Third, it was hard to compare the study results to other studies because data on program use are often very poorly reported or even completely lacking (for a review, see [20]). Furthermore, the study was subject to a considerable amount of attrition. This is a common finding in studies on eHealth interventions [20]. Dropout appeared higher, for example, among mothers with a lower educational level, which has also been reported for other web-based tailored interventions [76]. In this study, missing data and selective dropout were handled by using multiple imputation [55,56]. Fortunately, there were nearly no differences between the results from the complete case analyses and the ITT analyses. Therefore, it seems unlikely that the effects found are spurious or due to selective dropout. Finally, caution is needed when generalizing the results of this study to the general population (ie, Dutch mothers of 12-year-old girls) because we were unable to check the representativeness of the sample. Besides, it should be noted that a very homogenous sample was reached. An undiversified reach is a known phenomenon within web-based interventions aimed at health promotion [20]. In our study, the mothers were predominantly born in the Netherlands, highly educated, and likely to have a daughter that was vaccinated against HPV. However, results from the effect evaluation showed no differences in intervention effects in specific subgroups of participants, as indicated by moderation analyses with sociodemographic variables [30].

### Conclusions and Recommendations

This process evaluation has demonstrated that program use and acceptability of the web-based tailored HPV vaccination intervention were adequate. We found a positive association between completeness and acceptability. Furthermore, dose-response effects were found for completeness and time of website use on (nearly) all social-psychological determinants of the mothers’ decision making about vaccination, levels of IDM, and decisional conflict. Importantly, the extent to which mothers completed the intervention positively influenced their daughters’ actual HPV vaccination uptake. These results indicate that this web-based, tailored intervention fits well with the mothers’ needs and has the potential to increase HPV vaccination uptake. Because of the intervention’s adequate (dose-response) effects and acceptability, the intervention has recently been incorporated into the national HPV vaccination communication alongside the existing communication materials.

We recommend future interventions promoting HPV vaccination acceptance to (1) incorporate a process evaluation alongside the effect evaluation, (2), include strategies to arouse interest so as to expand reach, and to (3) include time on task. In addition, we recommend investigating the influence of different media types (eg, graphic vs nongraphic presentations) on decision making about HPV vaccination. For instance, Cox and colleagues [77] found that parents who viewed a graphic presentation of HPV-related risk information had a higher HPV vaccination intention compared to parents who viewed a nongraphic presentation. Furthermore, we recommend future research to examine differences in use and acceptability in specific subgroups of participants, for example by conducting moderation analyses with sociodemographics (eg, educational level). Finally, we recommend developing similar intervention models for (a) target groups other than mothers (eg, the daughter or sons in case they will become a next target group for HPV vaccination) and (b) other vaccinations (eg, maternal pertussis vaccination).

## Appendix

Multimedia Appendix 1 Screenshots of the website.

Multimedia Appendix 2 Measurement details (secondary outcomes).

## Abbreviations

eHealth: electronic health
HPV: human papilloma virus
IDM: informed decision making
ITT: intention-to-treat
OR: odds ratio
