# Supplementary material for: Dose-Response Relationship of a Web-Based Tailored Intervention Promoting Human Papillomavirus Vaccination: Process Evaluation of a Randomized Controlled Trial
Source: J Med Internet Res. 2020 Jul 17;22(7):e14822. doi: 10.2196/14822 (PMC7395256; doi:10.2196/14822)

**Multimedia Appendix 1**

Screenshots of the website

**
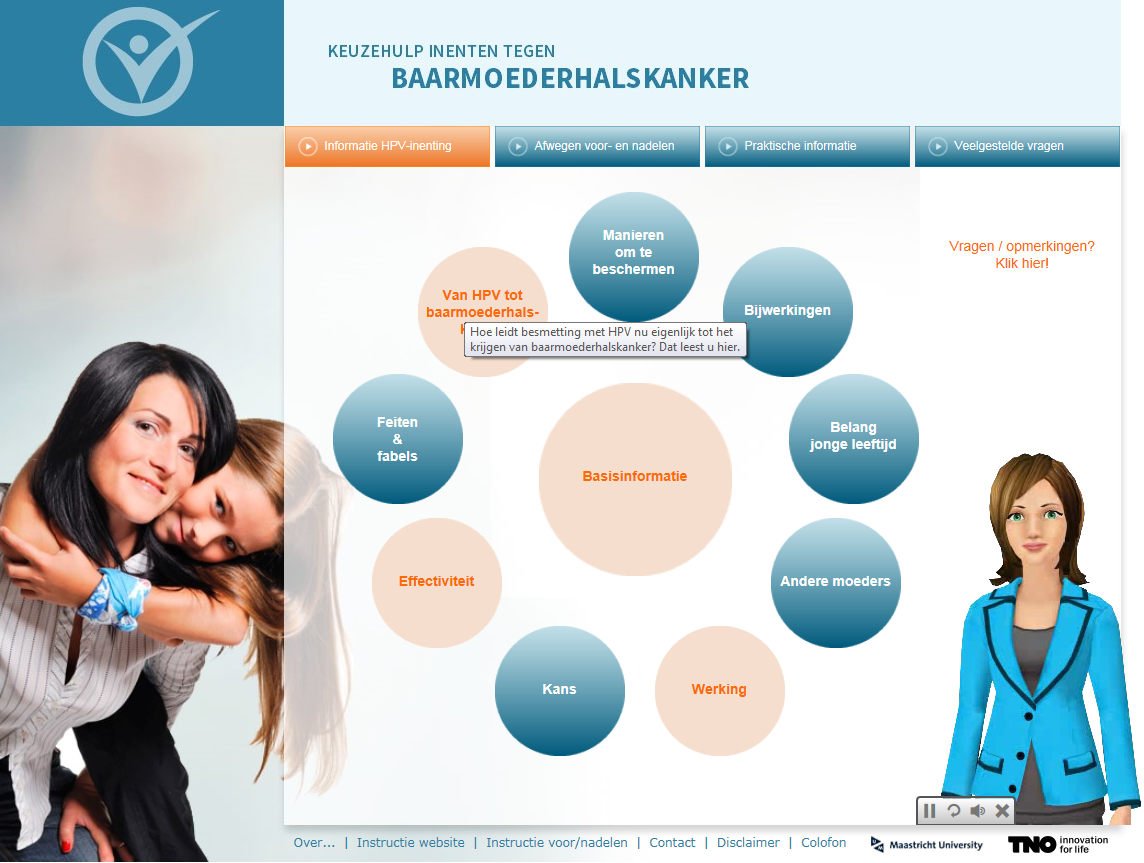
**Picture 1: The first menu of the website (‘information about the HPV-vaccination’).


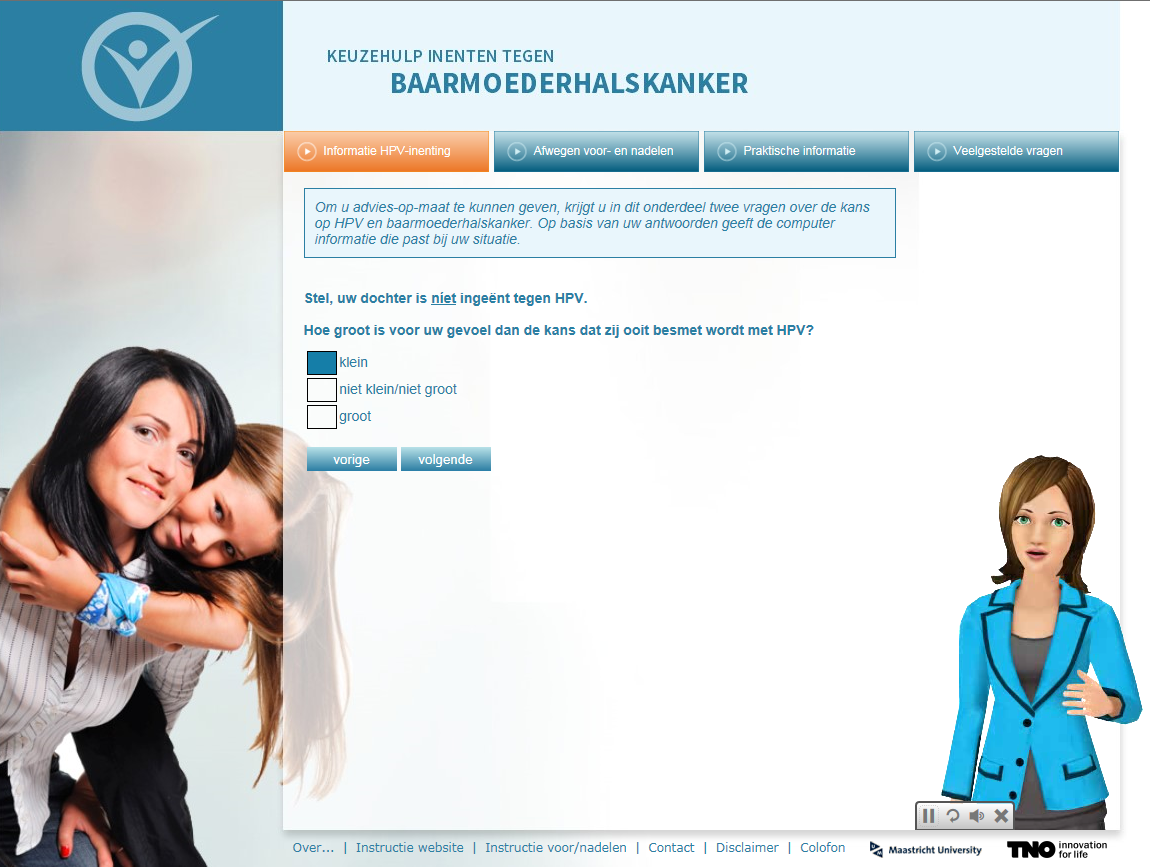
Picture 2: Within the ‘risk component’ of menu 1, the assistant asks a question about a mothers’ risk perception of her daughter getting infected with HPV on which feedback was then tailored (Picture 3).

….

Picture 3: Tailored feedback on mothers’ risk perception by the doctor-like assistant.


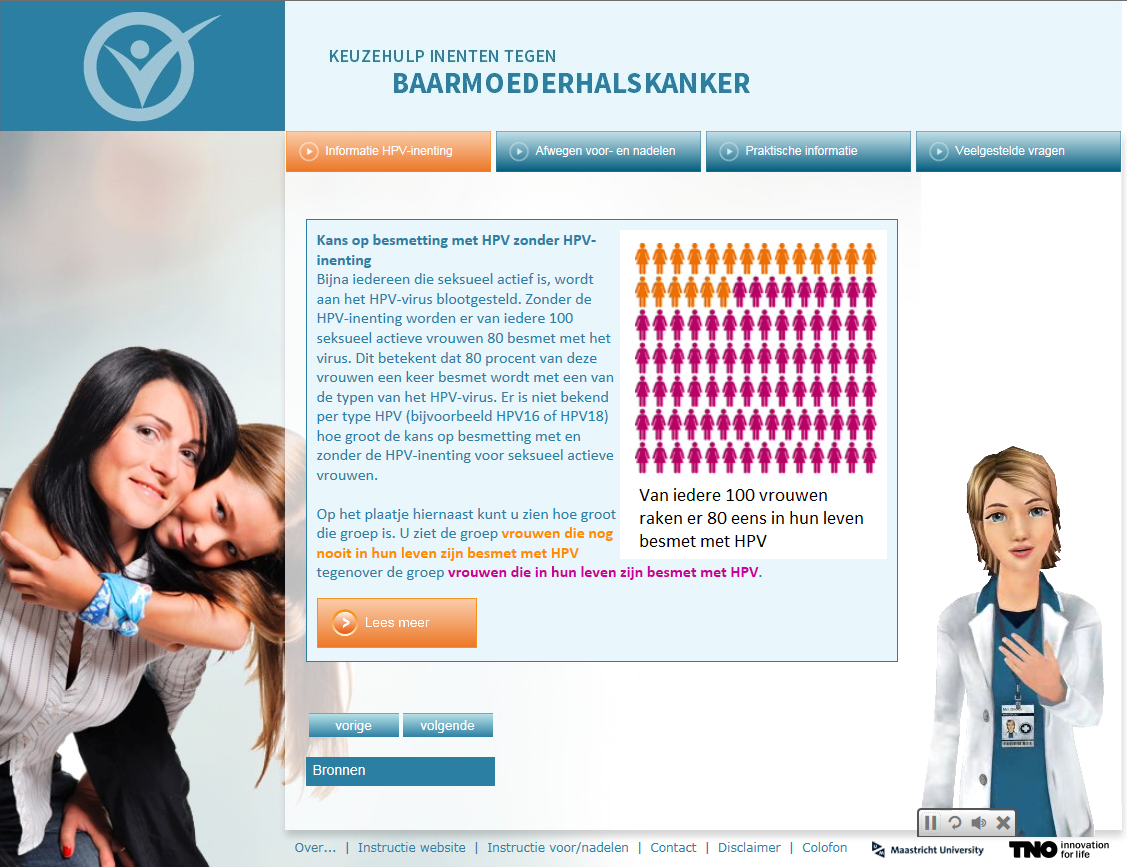


Picture 4: The third menu of the website (‘practical information’).


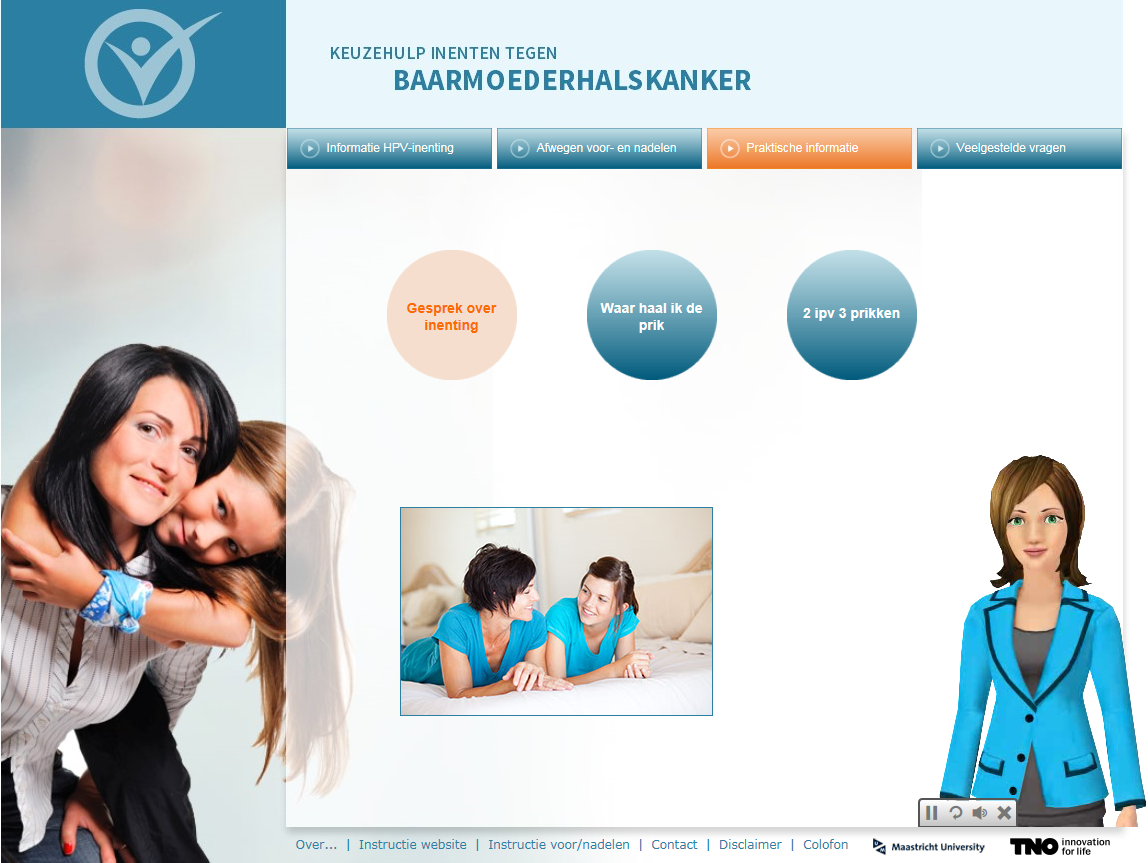


Picture 4: The fourth menu of the website (‘frequently asked questions’).


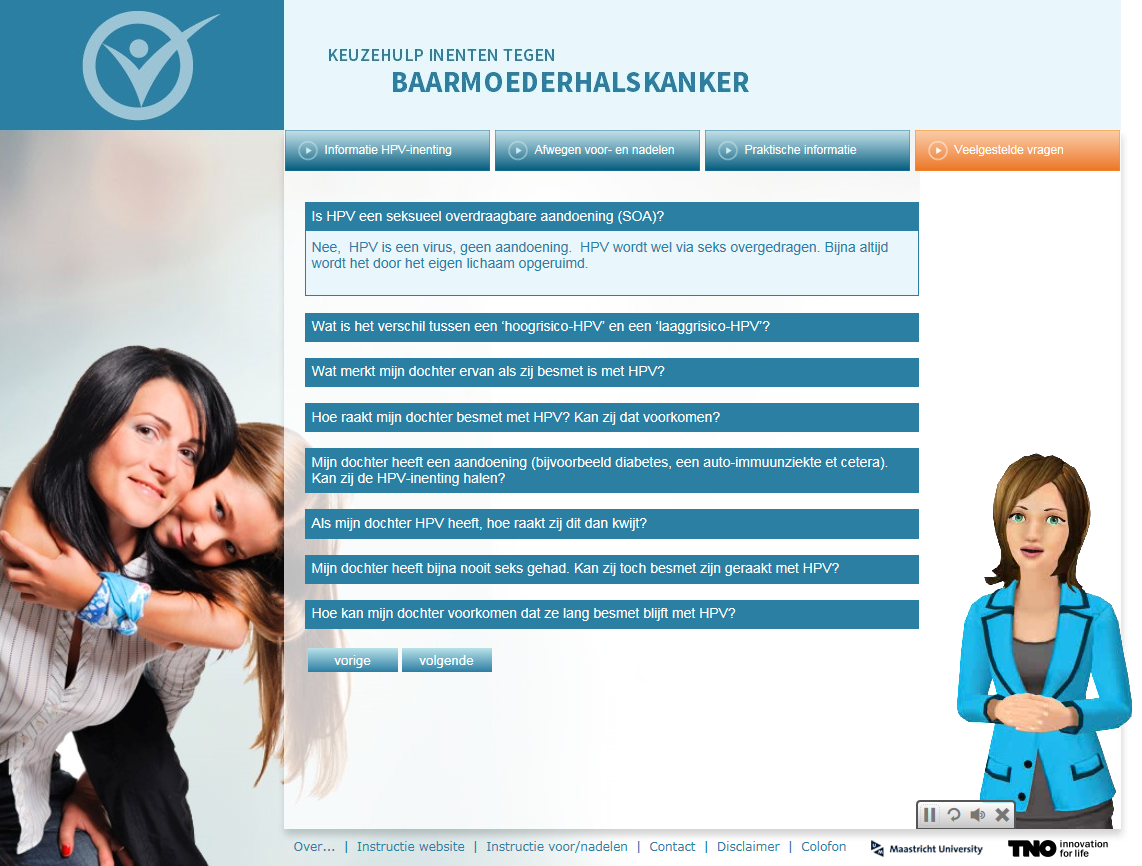

Supplement: Multimedia Appendix 1 [file jmir_v22i7e14822_app1.docx]
