# Supplementary material for: Dose-Response Relationship of a Web-Based Tailored Intervention Promoting Human Papillomavirus Vaccination: Process Evaluation of a Randomized Controlled Trial
Source: J Med Internet Res. 2020 Jul 17;22(7):e14822. doi: 10.2196/14822 (PMC7395256; doi:10.2196/14822)
Supplement: Multimedia Appendix 2 [file jmir_v22i7e14822_app2.doc]

**Multimedia Appendix 1: Measurement details (secondary outcomes)**

Informed decision-making (IDM) and decisional conflict

IDM was measured both dichotomously and continuously (see Table 1 below) [30]. Both measures were based on the Multi-dimensional Measure of Informed Choice (MMIC) [45-47].

The dichotomous outcome of IDM was classified as an informed decision if (1) mothers had sufficient knowledge and (2) there was consistency between their attitude and behavior (HPV vaccination uptake). To be sufficient, knowledge had to be higher than the baseline mean score. A decision was considered to be consistent when the mother’s attitude was positive (higher than 4 on a 7-point scale) and her daughter was vaccinated, or when her attitude was negative (lower than 4 on a 7-point scale) and her daughter was not vaccinated. All other combinations were classified as an uninformed decision.

The continuous measure of IDM was constructed by first recoding attitude from 0-7 to −3 (negative) to 3 (positive attitude) and HPV uptake from 0 or 1 to −1 (no injection) or 1 (1 or 2 injections). Next, level of consistency (−3 = low consistency; 3 = high consistency) was determined by multiplying the scores for attitude by those for HPV uptake. Consistency was then recoded into 0 (least consistent) to 6 (most consistent). Then, knowledge (-8 to 8) was recoded into 0 (least knowledgeable) to 8 (most knowledgeable; original scores below 0 were all recoded to 0. Finally, the continuous measure for IDM was computed by multiplying knowledge (0-8) with consistency (0-6). This resulted in a scale ranging from 0 (least informed) to 48 (most informed).

Decisional conflict was measured using the “Uncertainty” subscale of the Decisional Conflict Scales [48], which included 3 items on a 7-point scale (e.g., “as regards to the HPV vaccination, the decision was relatively easy to make) (1 = completely disagree to 7 = completely agree). Internal consistency was high (Cronbach α =.94).

Determinants of HPV-vaccination uptake

All scores on scaled items showed sufficient internal consistency (Cronbach α ≥.78/Pearson r ≥.64), hence were averaged into a scale. Table 1 provides an overview of the primary and secondary outcome measures. Items with an (R) were reverse coded.

Table 1. An overview of the primary and secondary outcome measures.

| **Measures** |  | **Items** | **Score/scale (minimum to maximum value)** | **Cronbach alpha (α) or Pearson *r* (*r*)^2^** | **References** |
| --- | --- | --- | --- | --- | --- |
| *Primary outcome* |  |  |  |  |  |
|  | HPV^a^ vaccination uptake | Uptake of the HPV vaccination is obtained through data from Praeventis. | 0 = not vaccinated  1 = vaccinated | N/A^b^ |  |
| *Secondary outcomes* |  |  |  |  |  |
|  | IDM^c^ outcome (dichotomous) | An informed decision has been made when:  the knowledge score was higher or equal to the mean of knowledge at baseline, the attitude score was higher than 4 (positive) and the HPV vaccination has been received. | 0 = not informed  1 = informed | N/A | [45-47] |
|  |  | The knowledge score was higher or equal to the mean of knowledge at baseline, the attitude score was lower than 4 (negative), and the HPV vaccination has not been received.  Any other combination was categorized as an uninformed decision. |  |  |  |
|  | IDM outcome (continuous) | Attitude was recoded from 0-7 to −3 (negative) to 3 (positive attitude) and HPV uptake was recoded from 0 or 1 to −1 (no injection) or 1 (1 or 2 injections).  Level of consistency was measured by multiplying the scores for attitude by those for HPV vaccination uptake (−3 = low consistency; 3 = high consistency). Consistency was then recoded into 0 (low) to 6 (high). Both consistency and sufficient knowledge were considered prerequisite for an informed decision.  Knowledge (-8 to 8) was recoded into 0 (least knowledgeable) to 8 (most knowledgeable); original scores below 0 were all recoded to 0.  The level of IDM outcome was determined by multiplying the scores for knowledge with those for consistency. | 0 = least informed decision to 48 = most informed decision | N/A | [30] |
|  | Decisional conflict about the HPV vaccination | As regards the HPV vaccination | 1 = high to 7 = low decisional conflict | .94 (α) | [48] |
|  |  | I felt sure about my choice |  |  |  |
|  |  | The decision was relatively easy to make |  |  |  |
|  |  | I was clear about the best choice for my daughter |  |  |  |
|  | HPV vaccination intention | Are you planning on getting your daughter vaccinated against HPV? | 1 = low intention to vaccinate to 7 = high intention to vaccinate | .92 (*r*) | [30,49,50] |
|  |  | How big is the chance that you will get your daughter vaccinated? |  |  |  |
|  | Attitude toward the HPV vaccination | Vaccinating my daughter against HPV is... | 1 = negative to 7 = positive attitude | .98 (α) | [51] |
|  |  | very undesirable to very desirable |  |  |  |
|  |  | very bad to very good |  |  |  |
|  |  | very negative to very positive |  |  |  |
|  |  | very unimportant to very important |  |  |  |
|  | Risk perception (having received no HPV vaccination) | Imagine that your daughter was not vaccinated against HPV.  The chance that my daughter will get cervical cancer is... | 1 = low to 7 = high risk perception | N/A | [51,52] |
|  | Risk perception (having received the HPV vaccination) | Imagine that your daughter was vaccinated against HPV.  The chance that my daughter will get cervical cancer is... | 1 = low to 7 = high risk perception | N/A | [51,52] |
|  | Anticipated regret about rejecting the HPV vaccination | Imagine your daughter has not received the HPV vaccination and she gets cervical cancer in the future.  How much would you regret your decision to let her receive no vaccination? | 1 = low to 7 = high anticipated regret | N/A | [30,49,50] |
|  | Beliefs about the HPV vaccination | If the government offers the vaccination, I assume it will be safe; | 1 = negative to 7 = positive beliefs about the HPV vaccination | .85 (α) | [52,53] |
|  |  | Our government shows responsibility for the health of the Dutch population by introducing the HPV vaccination |  |  |  |
|  |  | The HPV vaccination was only introduced because the pharmaceutical industry will earn a lot of money from it (R) |  |  |  |
|  |  | There is too little known about whether the HPV vaccination effectively protects against cervical cancer (R) |  |  |  |
|  |  | There is too little known about the detrimental side effects of the HPV vaccination (R) |  |  |  |
|  |  | My daughter is too young to receive the HPV vaccination (R) |  |  |  |
|  |  | My daughter does not need the vaccination because she is not yet sexually active (R) |  |  |  |
|  | Subjective norms toward the HPV vaccination^d^ | Normative beliefs: | −20 = negative to 20 = positive | .64 (*r*) | [51] |
|  |  | Regarding the HPV vaccination of your daughter, what is your expectation on the opinion of... |  |  |  |
|  |  | Social referents: partner^e^, daughter |  |  |  |
|  |  | Motivation to comply: |  |  |  |
|  |  | How motivated are you to comply with the opinion of...? |  |  |  |
|  | Habit strength toward the HPV vaccination | Letting my daughter receive the HPV vaccination is something I do | 1 = weak to 7 = strong habit strength | .78 (*r*) | [54] |
|  |  | automatically |  |  |  |
|  |  | without thinking |  |  |  |
|  | Self-efficacy expectations toward the HPV vaccination | To what extend would you succeed in dealing with the following statements? | 1 = low self-efficacy to 7 = high self-efficacy | .82 (α) | [30,49,50] |
|  |  | Guiding my daughter in the decision regarding the HPV vaccination |  |  |  |
|  |  | Having a good talk with my daughter about the HPV vaccination |  |  |  |
|  |  | Having a good talk with my partner^e^ about the HPV vaccination |  |  |  |
|  |  | Motivating my daughter to have herself vaccinated |  |  |  |
|  |  | Getting the actual HPV vaccination/2 injections with my daughter |  |  |  |
|  | Knowledge about the HPV vaccination^f^ | Are the following statements true or false? | −8 = incorrect, 8 = correct | N/A | [30,49,50] |
|  |  | HPV is sexually transmittable. |  |  |  |
|  |  | Condoms fully protect against HPV. |  |  |  |
|  |  | My daughter is obliged to get the HPV vaccination when she is invited. |  |  |  |
|  |  | You will always notice when you are infected by HPV. |  |  |  |
|  |  | Only women can get infected by HPV. |  |  |  |
|  |  | Women who received the HPV vaccination are still advised to participate in the cervical cancer screening in the Netherlands. |  |  |  |
|  |  | The HPV vaccination fully protects against cervical cancer. |  |  |  |
|  |  | My daughter does not need to get the HPV vaccination if she is already sexually active. |  |  |  |
|  | Relative effectiveness of the HPV vaccination^g^ | How would you rate the effectiveness of the following methods of preventing cervical cancer: | −9 = HPV vaccination least effective to 9 = HPV vaccination most effective | N/A | [30,49,50] |
|  |  | having safe sex |  |  |  |
|  |  | having sex with only 1 person in a lifetime |  |  |  |
|  |  | participating in the cervical cancer screening |  |  |  |
|  |  | having a healthy lifestyle (e.g., not smoking) |  |  |  |
|  |  | the HPV vaccination |  |  |  |
|  |  | Participants rated the effectiveness of each method |  |  |  |

^a^ HPV: human papillomavirus.

^b^ N/A: not applicable.

^c^ IDM: informed decision making.

^d^ The subjective norms score was first computed by multiplying normative beliefs and motivation to comply for each social referent, and then by summing up the multiplications of the social referents.

^e^ Only applicable if the mother indicated that she had a partner.

^f^ Knowledge is not a scale because the answer on 1 item does not predict the answer on other items; the items were summed up to present a sum score of knowledge.

^g^ The difference between the rated effectiveness of the HPV vaccination and the most effective alternative represented the relative effectiveness score (−9 = HPV vaccination least effective to 9 = HPV vaccination most effective).
